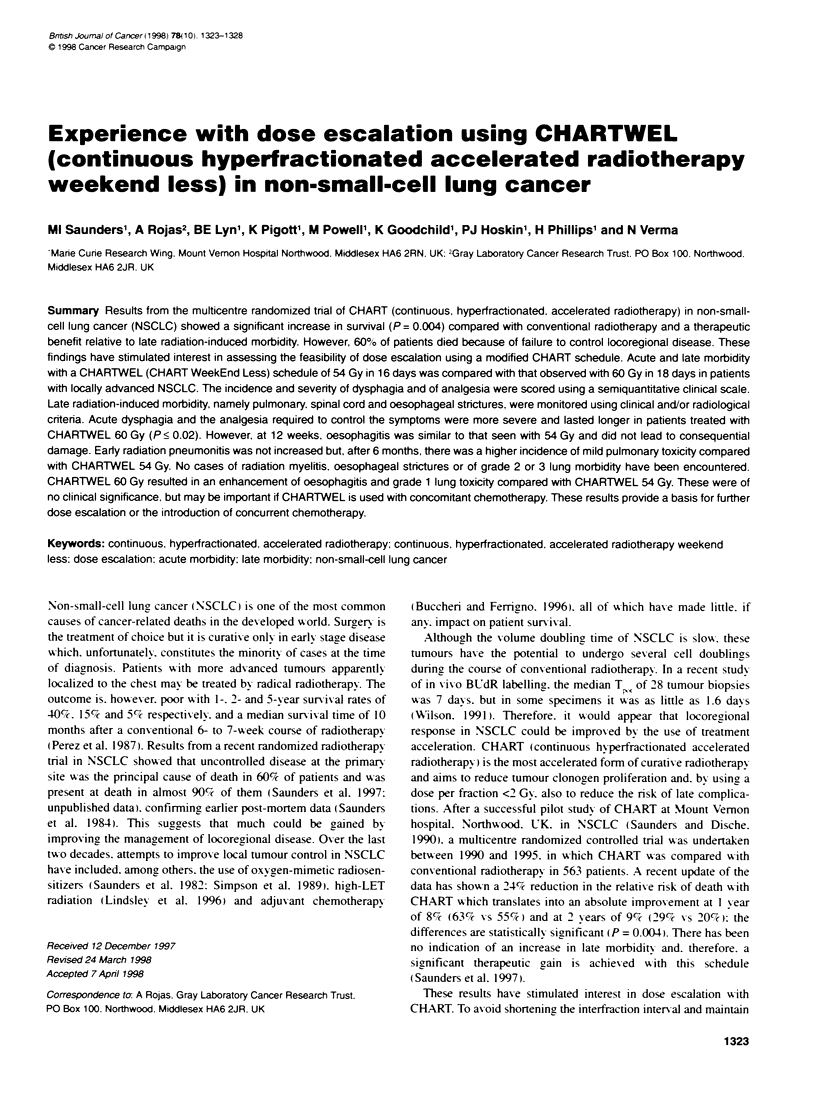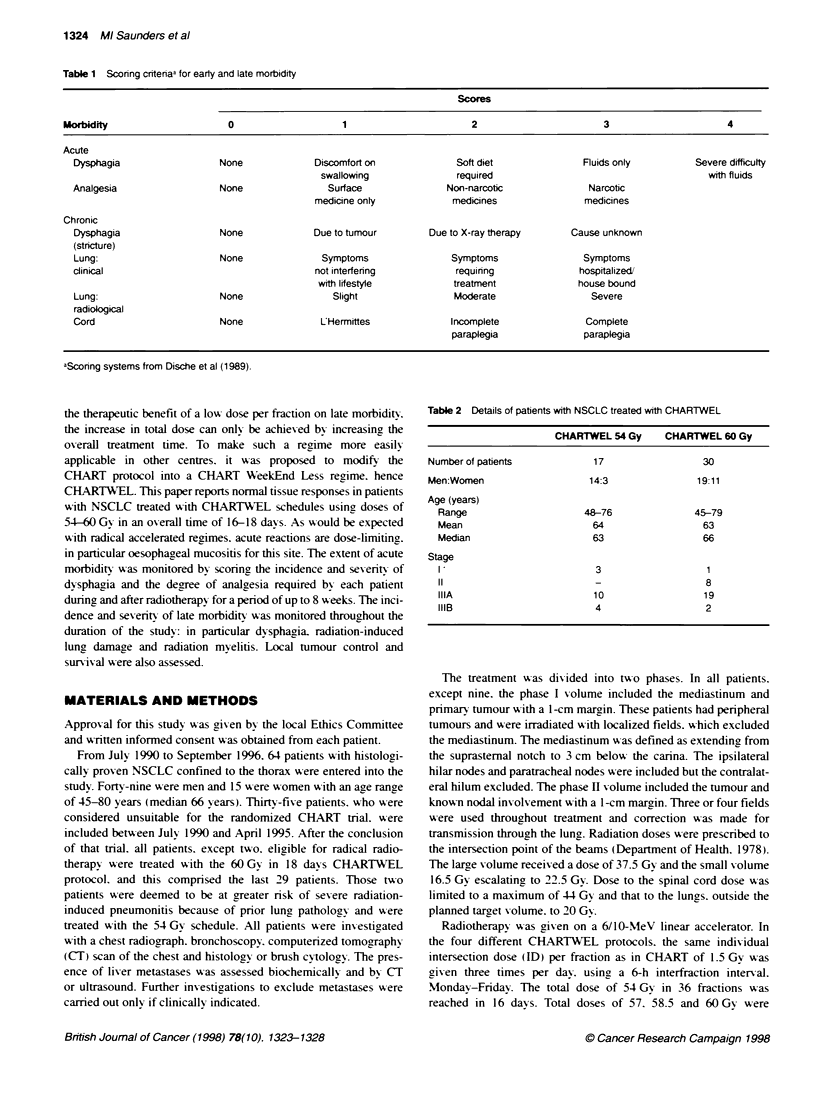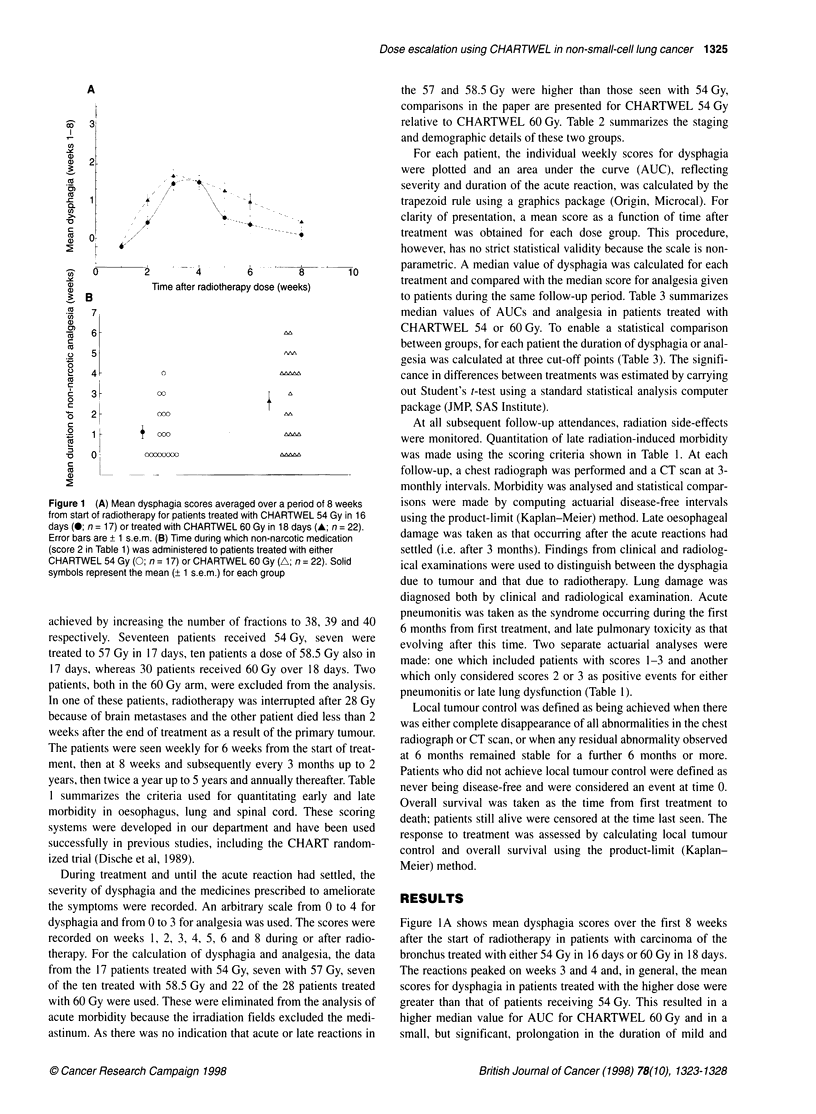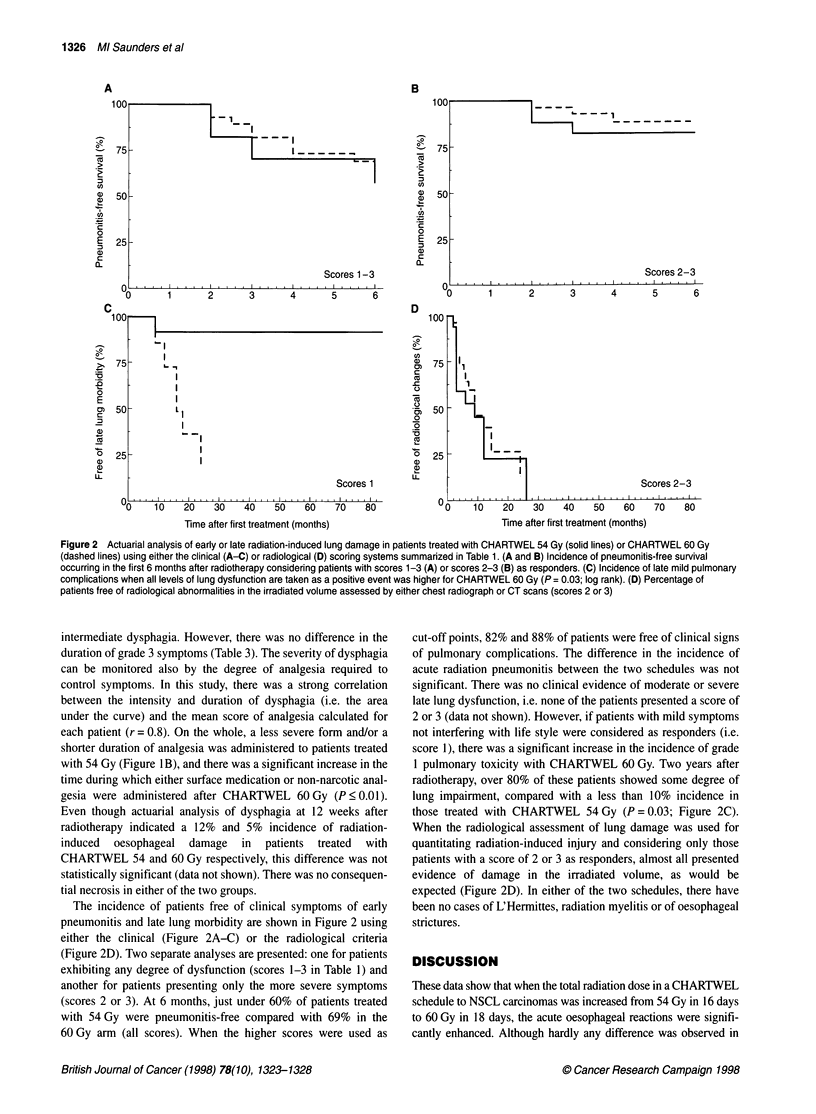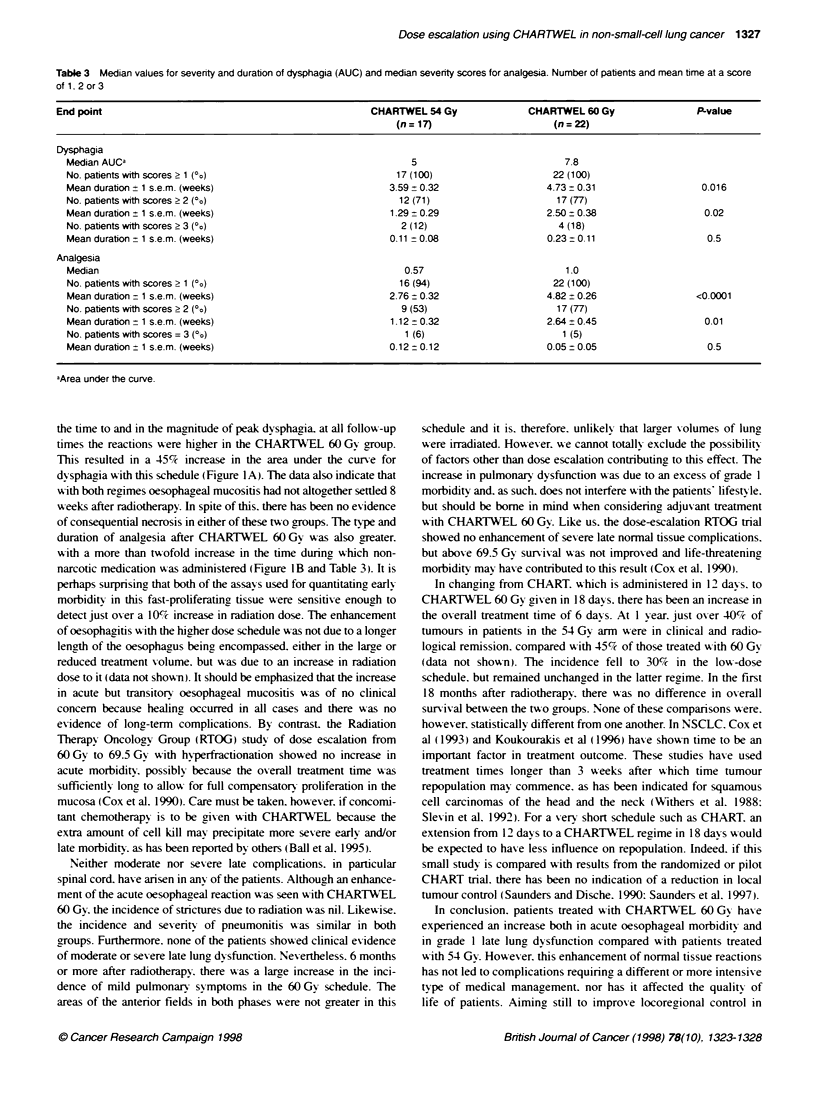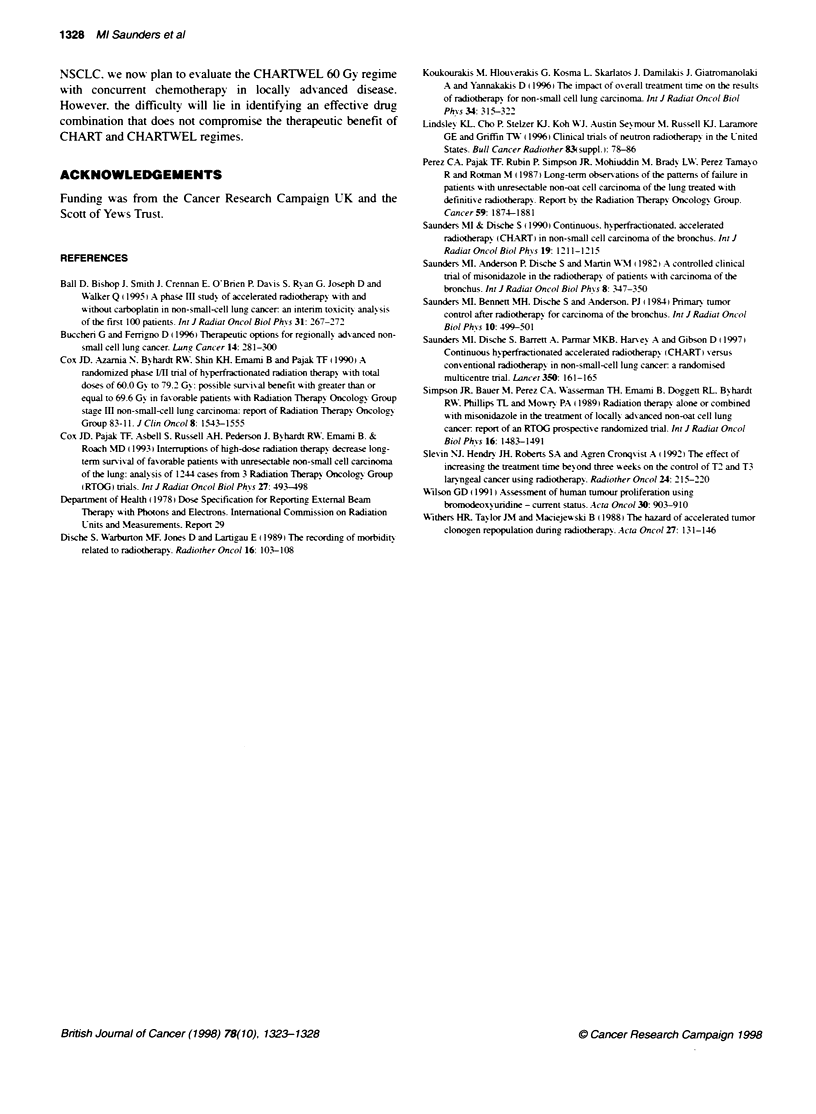# Experience with dose escalation using CHARTWEL (continuous hyperfractionated accelerated radiotherapy weekend less) in non-small-cell lung cancer.

**DOI:** 10.1038/bjc.1998.678

**Published:** 1998-11

**Authors:** M. I. Saunders, A. Rojas, B. E. Lyn, K. Pigott, M. Powell, K. Goodchild, P. J. Hoskin, H. Phillips, N. Verma

**Affiliations:** Marie Curie Research Wing, Mount Vernon Hospital Northwood, Middlesex, UK.

## Abstract

Results from the multicentre randomized trial of CHART (continuous, hyperfractionated, accelerated radiotherapy) in non-small-cell lung cancer (NSCLC) showed a significant increase in survival (P=0.004) compared with conventional radiotherapy and a therapeutic benefit relative to late radiation-induced morbidity. However, 60% of patients died because of failure to control locoregional disease. These findings have stimulated interest in assessing the feasibility of dose escalation using a modified CHART schedule. Acute and late morbidity with a CHARTWEL (CHART WeekEnd Less) schedule of 54 Gy in 16 days was compared with that observed with 60 Gy in 18 days in patients with locally advanced NSCLC. The incidence and severity of dysphagia and of analgesia were scored using a semiquantitative clinical scale. Late radiation-induced morbidity, namely pulmonary, spinal cord and oesophageal strictures, were monitored using clinical and/or radiological criteria. Acute dysphagia and the analgesia required to control the symptoms were more severe and lasted longer in patients treated with CHARTWEL 60 Gy (P< or = 0.02). However, at 12 weeks, oesophagitis was similar to that seen with 54 Gy and did not lead to consequential damage. Early radiation pneumonitis was not increased but, after 6 months, there was a higher incidence of mild pulmonary toxicity compared with CHARTWEL 54 Gy. No cases of radiation myelitis, oesophageal strictures or of grade 2 or 3 lung morbidity have been encountered. CHARTWEL 60 Gy resulted in an enhancement of oesophagitis and grade 1 lung toxicity compared with CHARTWEL 54 Gy. These were of no clinical significance, but may be important if CHARTWEL is used with concomitant chemotherapy. These results provide a basis for further dose escalation or the introduction of concurrent chemotherapy.


British Journal of Cancer (1998) 78(10), 1323–1328
© 1998 Cancer Research Campaign

### Experience with dose escalation using CHARTWEL (continuous hyperfractionated accelerated radiotherapy weekend less) in non-small-cell lung cancer

MI Saunders[1], A Rojas[2], BE Lyn[1], K Pigott[1], M Powell[1], K Goodchild[1], PJ Hoskin[1], H Phillips[1] and N Verma

[1]Marie Curie Research Wing, Mount Vernon Hospital Northwood, Middlesex HA6 2RN, UK; [2]Gray Laboratory Cancer Research Trust, PO Box 100, Northwood, Middlesex HA6 2JR, UK

**Summary** Results from the multicentre randomized trial of CHART (continuous, hyperfractionated, accelerated radiotherapy) in non-small-cell lung cancer (NSCLC) showed a significant increase in survival ($P = 0.004$) compared with conventional radiotherapy and a therapeutic benefit relative to late radiation-induced morbidity. However, 60% of patients died because of failure to control locoregional disease. These findings have stimulated interest in assessing the feasibility of dose escalation using a modified CHART schedule. Acute and late morbidity with a CHARTWEL (CHART WeekEnd Less) schedule of 54 Gy in 16 days was compared with that observed with 60 Gy in 18 days in patients with locally advanced NSCLC. The incidence and severity of dysphagia and of analgesia were scored using a semiquantitative clinical scale. Late radiation-induced morbidity, namely pulmonary, spinal cord and oesophageal strictures, were monitored using clinical and/or radiological criteria. Acute dysphagia and the analgesia required to control the symptoms were more severe and lasted longer in patients treated with CHARTWEL 60 Gy ($P \leq 0.02$). However, at 12 weeks, oesophagitis was similar to that seen with 54 Gy and did not lead to consequential damage. Early radiation pneumonitis was not increased but, after 6 months, there was a higher incidence of mild pulmonary toxicity compared with CHARTWEL 54 Gy. No cases of radiation myelitis, oesophageal strictures or of grade 2 or 3 lung morbidity have been encountered. CHARTWEL 60 Gy resulted in an enhancement of oesophagitis and grade 1 lung toxicity compared with CHARTWEL 54 Gy. These were of no clinical significance, but may be important if CHARTWEL is used with concomitant chemotherapy. These results provide a basis for further dose escalation or the introduction of concurrent chemotherapy.

**Keywords:** continuous, hyperfractionated, accelerated radiotherapy; continuous, hyperfractionated, accelerated radiotherapy weekend less; dose escalation; acute morbidity; late morbidity; non-small-cell lung cancer

Non-small-cell lung cancer (NSCLC) is one of the most common causes of cancer-related deaths in the developed world. Surgery is the treatment of choice but it is curative only in early stage disease which, unfortunately, constitutes the minority of cases at the time of diagnosis. Patients with more advanced tumours apparently localized to the chest may be treated by radical radiotherapy. The outcome is, however, poor with 1-, 2- and 5-year survival rates of 40%, 15% and 5% respectively, and a median survival time of 10 months after a conventional 6- to 7-week course of radiotherapy (Perez et al, 1987). Results from a recent randomized radiotherapy trial in NSCLC showed that uncontrolled disease at the primary site was the principal cause of death in 60% of patients and was present at death in almost 90% of them (Saunders et al, 1997; unpublished data), confirming earlier post-mortem data (Saunders et al, 1984). This suggests that much could be gained by improving the management of locoregional disease. Over the last two decades, attempts to improve local tumour control in NSCLC have included, among others, the use of oxygen-mimetic radiosensitizers (Saunders et al, 1982; Simpson et al, 1989), high-LET radiation (Lindsley et al, 1996) and adjuvant chemotherapy (Buccheri and Ferrigno, 1996), all of which have made little, if any, impact on patient survival.

Although the volume doubling time of NSCLC is slow, these tumours have the potential to undergo several cell doublings during the course of conventional radiotherapy. In a recent study of in vivo BUdR labelling, the median $T_{pot}$ of 28 tumour biopsies was 7 days, but in some specimens it was as little as 1.6 days (Wilson, 1991). Therefore, it would appear that locoregional response in NSCLC could be improved by the use of treatment acceleration. CHART (continuous hyperfractionated accelerated radiotherapy) is the most accelerated form of curative radiotherapy and aims to reduce tumour clonogen proliferation and, by using a dose per fraction <2 Gy, also to reduce the risk of late complications. After a successful pilot study of CHART at Mount Vernon hospital, Northwood, UK, in NSCLC (Saunders and Dische, 1990), a multicentre randomized controlled trial was undertaken between 1990 and 1995, in which CHART was compared with conventional radiotherapy in 563 patients. A recent update of the data has shown a 24% reduction in the relative risk of death with CHART which translates into an absolute improvement at 1 year of 8% (63% vs 55%) and at 2 years of 9% (29% vs 20%); the differences are statistically significant ($P = 0.004$). There has been no indication of an increase in late morbidity and, therefore, a significant therapeutic gain is achieved with this schedule (Saunders et al, 1997).

These results have stimulated interest in dose escalation with CHART. To avoid shortening the interfraction interval and maintain

*Received 12 December 1997*
*Revised 24 March 1998*
*Accepted 7 April 1998*

*Correspondence to:* A Rojas, Gray Laboratory Cancer Research Trust, PO Box 100, Northwood, Middlesex HA6 2JR, UK

**Table 1** Scoring criteria[a] for early and late morbidity

| | Scores | | | | |
|---|---|---|---|---|---|
| **Morbidity** | **0** | **1** | **2** | **3** | **4** |
| Acute | | | | | |
| Dysphagia | None | Discomfort on swallowing | Soft diet required | Fluids only | Severe difficulty with fluids |
| Analgesia | None | Surface medicine only | Non-narcotic medicines | Narcotic medicines | |
| Chronic | | | | | |
| Dysphagia (stricture) | None | Due to tumour | Due to X-ray therapy | Cause unknown | |
| Lung: clinical | None | Symptoms not interfering with lifestyle | Symptoms requiring treatment | Symptoms hospitalized/ house bound | |
| Lung: radiological | None | Slight | Moderate | Severe | |
| Cord | None | L'Hermittes | Incomplete paraplegia | Complete paraplegia | |

[a]Scoring systems from Dische et al (1989).

the therapeutic benefit of a low dose per fraction on late morbidity. the increase in total dose can only be achieved by increasing the overall treatment time. To make such a regime more easily applicable in other centres. it was proposed to modify the CHART protocol into a CHART WeekEnd Less regime. hence CHARTWEL. This paper reports normal tissue responses in patients with NSCLC treated with CHARTWEL schedules using doses of 54–60 Gy in an overall time of 16–18 days. As would be expected with radical accelerated regimes. acute reactions are dose-limiting. in particular oesophageal mucositis for this site. The extent of acute morbidity was monitored by scoring the incidence and severity of dysphagia and the degree of analgesia required by each patient during and after radiotherapy for a period of up to 8 weeks. The incidence and severity of late morbidity was monitored throughout the duration of the study: in particular dysphagia. radiation-induced lung damage and radiation myelitis. Local tumour control and survival were also assessed.

### MATERIALS AND METHODS

Approval for this study was given by the local Ethics Committee and written informed consent was obtained from each patient.

From July 1990 to September 1996. 64 patients with histologically proven NSCLC confined to the thorax were entered into the study. Forty-nine were men and 15 were women with an age range of 45–80 years (median 66 years). Thirty-five patients. who were considered unsuitable for the randomized CHART trial. were included between July 1990 and April 1995. After the conclusion of that trial. all patients. except two. eligible for radical radiotherapy were treated with the 60 Gy in 18 days CHARTWEL protocol. and this comprised the last 29 patients. Those two patients were deemed to be at greater risk of severe radiation-induced pneumonitis because of prior lung pathology and were treated with the 54 Gy schedule. All patients were investigated with a chest radiograph. bronchoscopy. computerized tomography (CT) scan of the chest and histology or brush cytology. The presence of liver metastases was assessed biochemically and by CT or ultrasound. Further investigations to exclude metastases were carried out only if clinically indicated.

**Table 2** Details of patients with NSCLC treated with CHARTWEL

| | CHARTWEL 54 Gy | CHARTWEL 60 Gy |
|---|---|---|
| Number of patients | 17 | 30 |
| Men:Women | 14:3 | 19:11 |
| Age (years) | | |
| Range | 48–76 | 45–79 |
| Mean | 64 | 63 |
| Median | 63 | 66 |
| Stage | | |
| I | 3 | 1 |
| II | – | 8 |
| IIIA | 10 | 19 |
| IIIB | 4 | 2 |

The treatment was divided into two phases. In all patients. except nine. the phase I volume included the mediastinum and primary tumour with a 1-cm margin. These patients had peripheral tumours and were irradiated with localized fields. which excluded the mediastinum. The mediastinum was defined as extending from the suprasternal notch to 3 cm below the carina. The ipsilateral hilar nodes and paratracheal nodes were included but the contralateral hilum excluded. The phase II volume included the tumour and known nodal involvement with a 1-cm margin. Three or four fields were used throughout treatment and correction was made for transmission through the lung. Radiation doses were prescribed to the intersection point of the beams (Department of Health. 1978). The large volume received a dose of 37.5 Gy and the small volume 16.5 Gy escalating to 22.5 Gy. Dose to the spinal cord dose was limited to a maximum of 44 Gy and that to the lungs. outside the planned target volume. to 20 Gy.

Radiotherapy was given on a 6/10-MeV linear accelerator. In the four different CHARTWEL protocols. the same individual intersection dose (ID) per fraction as in CHART of 1.5 Gy was given three times per day. using a 6-h interfraction interval. Monday–Friday. The total dose of 54 Gy in 36 fractions was reached in 16 days. Total doses of 57. 58.5 and 60 Gy were

 © Cancer Research Campaign 1998

**Figure 1** (**A**) Mean dysphagia scores averaged over a period of 8 weeks from start of radiotherapy for patients treated with CHARTWEL 54 Gy in 16 days (●; $n = 17$) or treated with CHARTWEL 60 Gy in 18 days (▲; $n = 22$). Error bars are ± 1 s.e.m. (**B**) Time during which non-narcotic medication (score 2 in Table 1) was administered to patients treated with either CHARTWEL 54 Gy (○; $n = 17$) or CHARTWEL 60 Gy (△; $n = 22$). Solid symbols represent the mean (± 1 s.e.m.) for each group achieved by increasing the number of fractions to 38, 39 and 40 respectively. Seventeen patients received 54 Gy, seven were treated to 57 Gy in 17 days, ten patients a dose of 58.5 Gy also in 17 days, whereas 30 patients received 60 Gy over 18 days. Two patients, both in the 60 Gy arm, were excluded from the analysis. In one of these patients, radiotherapy was interrupted after 28 Gy because of brain metastases and the other patient died less than 2 weeks after the end of treatment as a result of the primary tumour. The patients were seen weekly for 6 weeks from the start of treatment, then at 8 weeks and subsequently every 3 months up to 2 years, then twice a year up to 5 years and annually thereafter. Table 1 summarizes the criteria used for quantitating early and late morbidity in oesophagus, lung and spinal cord. These scoring systems were developed in our department and have been used successfully in previous studies, including the CHART randomized trial (Dische et al, 1989).

During treatment and until the acute reaction had settled, the severity of dysphagia and the medicines prescribed to ameliorate the symptoms were recorded. An arbitrary scale from 0 to 4 for dysphagia and from 0 to 3 for analgesia was used. The scores were recorded on weeks 1, 2, 3, 4, 5, 6 and 8 during or after radiotherapy. For the calculation of dysphagia and analgesia, the data from the 17 patients treated with 54 Gy, seven with 57 Gy, seven of the ten treated with 58.5 Gy and 22 of the 28 patients treated with 60 Gy were used. These were eliminated from the analysis of acute morbidity because the irradiation fields excluded the mediastinum. As there was no indication that acute or late reactions in the 57 and 58.5 Gy were higher than those seen with 54 Gy, comparisons in the paper are presented for CHARTWEL 54 Gy relative to CHARTWEL 60 Gy. Table 2 summarizes the staging and demographic details of these two groups.

For each patient, the individual weekly scores for dysphagia were plotted and an area under the curve (AUC), reflecting severity and duration of the acute reaction, was calculated by the trapezoid rule using a graphics package (Origin, Microcal). For clarity of presentation, a mean score as a function of time after treatment was obtained for each dose group. This procedure, however, has no strict statistical validity because the scale is non-parametric. A median value of dysphagia was calculated for each treatment and compared with the median score for analgesia given to patients during the same follow-up period. Table 3 summarizes median values of AUCs and analgesia in patients treated with CHARTWEL 54 or 60 Gy. To enable a statistical comparison between groups, for each patient the duration of dysphagia or analgesia was calculated at three cut-off points (Table 3). The significance in differences between treatments was estimated by carrying out Student's *t*-test using a standard statistical analysis computer package (JMP, SAS Institute).

At all subsequent follow-up attendances, radiation side-effects were monitored. Quantitation of late radiation-induced morbidity was made using the scoring criteria shown in Table 1. At each follow-up, a chest radiograph was performed and a CT scan at 3-monthly intervals. Morbidity was analysed and statistical comparisons were made by computing actuarial disease-free intervals using the product-limit (Kaplan–Meier) method. Late oesophageal damage was taken as that occurring after the acute reactions had settled (i.e. after 3 months). Findings from clinical and radiological examinations were used to distinguish between the dysphagia due to tumour and that due to radiotherapy. Lung damage was diagnosed both by clinical and radiological examination. Acute pneumonitis was taken as the syndrome occurring during the first 6 months from first treatment, and late pulmonary toxicity as that evolving after this time. Two separate actuarial analyses were made: one which included patients with scores 1–3 and another which only considered scores 2 or 3 as positive events for either pneumonitis or late lung dysfunction (Table 1).

Local tumour control was defined as being achieved when there was either complete disappearance of all abnormalities in the chest radiograph or CT scan, or when any residual abnormality observed at 6 months remained stable for a further 6 months or more. Patients who did not achieve local tumour control were defined as never being disease-free and were considered an event at time 0. Overall survival was taken as the time from first treatment to death; patients still alive were censored at the time last seen. The response to treatment was assessed by calculating local tumour control and overall survival using the product-limit (Kaplan–Meier) method.

### RESULTS

Figure 1A shows mean dysphagia scores over the first 8 weeks after the start of radiotherapy in patients with carcinoma of the bronchus treated with either 54 Gy in 16 days or 60 Gy in 18 days. The reactions peaked on weeks 3 and 4 and, in general, the mean scores for dysphagia in patients treated with the higher dose were greater than that of patients receiving 54 Gy. This resulted in a higher median value for AUC for CHARTWEL 60 Gy and in a small, but significant, prolongation in the duration of mild and

© Cancer Research Campaign 1998

**Figure 2** Actuarial analysis of early or late radiation-induced lung damage in patients treated with CHARTWEL 54 Gy (solid lines) or CHARTWEL 60 Gy (dashed lines) using either the clinical (**A–C**) or radiological (**D**) scoring systems summarized in Table 1. (**A** and **B**) Incidence of pneumonitis-free survival occurring in the first 6 months after radiotherapy considering patients with scores 1–3 (**A**) or scores 2–3 (**B**) as responders. (**C**) Incidence of late mild pulmonary complications when all levels of lung dysfunction are taken as a positive event was higher for CHARTWEL 60 Gy ($P = 0.03$; log rank). (**D**) Percentage of patients free of radiological abnormalities in the irradiated volume assessed by either chest radiograph or CT scans (scores 2 or 3)

intermediate dysphagia. However, there was no difference in the duration of grade 3 symptoms (Table 3). The severity of dysphagia can be monitored also by the degree of analgesia required to control symptoms. In this study, there was a strong correlation between the intensity and duration of dysphagia (i.e. the area under the curve) and the mean score of analgesia calculated for each patient ($r = 0.8$). On the whole, a less severe form and/or a shorter duration of analgesia was administered to patients treated with 54 Gy (Figure 1B), and there was a significant increase in the time during which either surface medication or non-narcotic analgesia were administered after CHARTWEL 60 Gy ($P \leq 0.01$). Even though actuarial analysis of dysphagia at 12 weeks after radiotherapy indicated a 12% and 5% incidence of radiation-induced oesophageal damage in patients treated with CHARTWEL 54 and 60 Gy respectively, this difference was not statistically significant (data not shown). There was no consequential necrosis in either of the two groups.

The incidence of patients free of clinical symptoms of early pneumonitis and late lung morbidity are shown in Figure 2 using either the clinical (Figure 2A–C) or the radiological criteria (Figure 2D). Two separate analyses are presented: one for patients exhibiting any degree of dysfunction (scores 1–3 in Table 1) and another for patients presenting only the more severe symptoms (scores 2 or 3). At 6 months, just under 60% of patients treated with 54 Gy were pneumonitis-free compared with 69% in the 60 Gy arm (all scores). When the higher scores were used as cut-off points, 82% and 88% of patients were free of clinical signs of pulmonary complications. The difference in the incidence of acute radiation pneumonitis between the two schedules was not significant. There was no clinical evidence of moderate or severe late lung dysfunction, i.e. none of the patients presented a score of 2 or 3 (data not shown). However, if patients with mild symptoms not interfering with life style were considered as responders (i.e. score 1), there was a significant increase in the incidence of grade 1 pulmonary toxicity with CHARTWEL 60 Gy. Two years after radiotherapy, over 80% of these patients showed some degree of lung impairment, compared with a less than 10% incidence in those treated with CHARTWEL 54 Gy ($P = 0.03$; Figure 2C). When the radiological assessment of lung damage was used for quantitating radiation-induced injury and considering only those patients with a score of 2 or 3 as responders, almost all presented evidence of damage in the irradiated volume, as would be expected (Figure 2D). In either of the two schedules, there have been no cases of L'Hermittes, radiation myelitis or of oesophageal strictures.

### DISCUSSION

These data show that when the total radiation dose in a CHARTWEL schedule to NSCL carcinomas was increased from 54 Gy in 16 days to 60 Gy in 18 days, the acute oesophageal reactions were significantly enhanced. Although hardly any difference was observed in

© Cancer Research Campaign 1998

**Table 3** Median values for severity and duration of dysphagia (AUC) and median severity scores for analgesia. Number of patients and mean time at a score of 1, 2 or 3

| End point | CHARTWEL 54 Gy ($n$ = 17) | CHARTWEL 60 Gy ($n$ = 22) | $P$-value |
|---|---|---|---|
| Dysphagia | | | |
| Median AUC[a] | 5 | 7.8 | |
| No. patients with scores ≥ 1 (%) | 17 (100) | 22 (100) | |
| Mean duration ± 1 s.e.m. (weeks) | 3.59 ± 0.32 | 4.73 ± 0.31 | 0.016 |
| No. patients with scores ≥ 2 (%) | 12 (71) | 17 (77) | |
| Mean duration ± 1 s.e.m. (weeks) | 1.29 ± 0.29 | 2.50 ± 0.38 | 0.02 |
| No. patients with scores ≥ 3 (%) | 2 (12) | 4 (18) | |
| Mean duration ± 1 s.e.m. (weeks) | 0.11 ± 0.08 | 0.23 ± 0.11 | 0.5 |
| Analgesia | | | |
| Median | 0.57 | 1.0 | |
| No. patients with scores ≥ 1 (%) | 16 (94) | 22 (100) | |
| Mean duration ± 1 s.e.m. (weeks) | 2.76 ± 0.32 | 4.82 ± 0.26 | <0.0001 |
| No. patients with scores ≥ 2 (%) | 9 (53) | 17 (77) | |
| Mean duration ± 1 s.e.m. (weeks) | 1.12 ± 0.32 | 2.64 ± 0.45 | 0.01 |
| No. patients with scores = 3 (%) | 1 (6) | 1 (5) | |
| Mean duration ± 1 s.e.m. (weeks) | 0.12 ± 0.12 | 0.05 ± 0.05 | 0.5 |

[a]Area under the curve.

the time to and in the magnitude of peak dysphagia, at all follow-up times the reactions were higher in the CHARTWEL 60 Gy group. This resulted in a 45% increase in the area under the curve for dysphagia with this schedule (Figure 1A). The data also indicate that with both regimes oesophageal mucositis had not altogether settled 8 weeks after radiotherapy. In spite of this, there has been no evidence of consequential necrosis in either of these two groups. The type and duration of analgesia after CHARTWEL 60 Gy was also greater, with a more than twofold increase in the time during which non-narcotic medication was administered (Figure 1B and Table 3). It is perhaps surprising that both of the assays used for quantitating early morbidity in this fast-proliferating tissue were sensitive enough to detect just over a 10% increase in radiation dose. The enhancement of oesophagitis with the higher dose schedule was not due to a longer length of the oesophagus being encompassed, either in the large or reduced treatment volume, but was due to an increase in radiation dose to it (data not shown). It should be emphasized that the increase in acute but transitory oesophageal mucositis was of no clinical concern because healing occurred in all cases and there was no evidence of long-term complications. By contrast, the Radiation Therapy Oncology Group (RTOG) study of dose escalation from 60 Gy to 69.5 Gy with hyperfractionation showed no increase in acute morbidity, possibly because the overall treatment time was sufficiently long to allow for full compensatory proliferation in the mucosa (Cox et al, 1990). Care must be taken, however, if concomitant chemotherapy is to be given with CHARTWEL because the extra amount of cell kill may precipitate more severe early and/or late morbidity, as has been reported by others (Ball et al, 1995).

Neither moderate nor severe late complications, in particular spinal cord, have arisen in any of the patients. Although an enhancement of the acute oesophageal reaction was seen with CHARTWEL 60 Gy, the incidence of strictures due to radiation was nil. Likewise, the incidence and severity of pneumonitis was similar in both groups. Furthermore, none of the patients showed clinical evidence of moderate or severe late lung dysfunction. Nevertheless, 6 months or more after radiotherapy, there was a large increase in the incidence of mild pulmonary symptoms in the 60 Gy schedule. The areas of the anterior fields in both phases were not greater in this schedule and it is, therefore, unlikely that larger volumes of lung were irradiated. However, we cannot totally exclude the possibility of factors other than dose escalation contributing to this effect. The increase in pulmonary dysfunction was due to an excess of grade 1 morbidity and, as such, does not interfere with the patients' lifestyle, but should be borne in mind when considering adjuvant treatment with CHARTWEL 60 Gy. Like us, the dose-escalation RTOG trial showed no enhancement of severe late normal tissue complications, but above 69.5 Gy survival was not improved and life-threatening morbidity may have contributed to this result (Cox et al, 1990).

In changing from CHART, which is administered in 12 days, to CHARTWEL 60 Gy given in 18 days, there has been an increase in the overall treatment time of 6 days. At 1 year, just over 40% of tumours in patients in the 54 Gy arm were in clinical and radiological remission, compared with 45% of those treated with 60 Gy (data not shown). The incidence fell to 30% in the low-dose schedule, but remained unchanged in the latter regime. In the first 18 months after radiotherapy, there was no difference in overall survival between the two groups. None of these comparisons were, however, statistically different from one another. In NSCLC, Cox et al (1993) and Koukourakis et al (1996) have shown time to be an important factor in treatment outcome. These studies have used treatment times longer than 3 weeks after which time tumour repopulation may commence, as has been indicated for squamous cell carcinomas of the head and the neck (Withers et al, 1988; Slevin et al, 1992). For a very short schedule such as CHART, an extension from 12 days to a CHARTWEL regime in 18 days would be expected to have less influence on repopulation. Indeed, if this small study is compared with results from the randomized or pilot CHART trial, there has been no indication of a reduction in local tumour control (Saunders and Dische, 1990; Saunders et al, 1997).

In conclusion, patients treated with CHARTWEL 60 Gy have experienced an increase both in acute oesophageal morbidity and in grade 1 late lung dysfunction compared with patients treated with 54 Gy. However, this enhancement of normal tissue reactions has not led to complications requiring a different or more intensive type of medical management, nor has it affected the quality of life of patients. Aiming still to improve locoregional control in

© Cancer Research Campaign 1998

NSCLC, we now plan to evaluate the CHARTWEL 60 Gy regime with concurrent chemotherapy in locally advanced disease. However, the difficulty will lie in identifying an effective drug combination that does not compromise the therapeutic benefit of CHART and CHARTWEL regimes.

### ACKNOWLEDGEMENTS

Funding was from the Cancer Research Campaign UK and the Scott of Yews Trust.

### REFERENCES

Ball D, Bishop J, Smith J, Crennan E, O'Brien P, Davis S, Ryan G, Joseph D and Walker Q (1995) A phase III study of accelerated radiotherapy with and without carboplatin in non-small-cell lung cancer: an interim toxicity analysis of the first 100 patients. *Int J Radiat Oncol Biol Phys* **31**: 267–272

Buccheri G and Ferrigno D (1996) Therapeutic options for regionally advanced non-small cell lung cancer. *Lung Cancer* **14**: 281–300

Cox JD, Azarnia N, Byhardt RW, Shin KH, Emami B and Pajak TF (1990) A randomized phase I/II trial of hyperfractionated radiation therapy with total doses of 60.0 Gy to 79.2 Gy: possible survival benefit with greater than or equal to 69.6 Gy in favorable patients with Radiation Therapy Oncology Group stage III non-small-cell lung carcinoma: report of Radiation Therapy Oncology Group 83-11. *J Clin Oncol* **8**: 1543–1555

Cox JD, Pajak TF, Asbell S, Russell AH, Pederson J, Byhardt RW, Emami B, & Roach MD (1993) Interruptions of high-dose radiation therapy decrease long-term survival of favorable patients with unresectable non-small cell carcinoma of the lung: analysis of 1244 cases from 3 Radiation Therapy Oncology Group (RTOG) trials. *Int J Radiat Oncol Biol Phys* **27**: 493–498

Department of Health (1978) Dose Specification for Reporting External Beam Therapy with Photons and Electrons. International Commission on Radiation Units and Measurements, Report 29

Dische S, Warburton MF, Jones D and Lartigau E (1989) The recording of morbidity related to radiotherapy. *Radiother Oncol* **16**: 103–108

Koukourakis M, Hlouverakis G, Kosma L, Skarlatos J, Damilakis J, Giatromanolaki A and Yannakakis D (1996) The impact of overall treatment time on the results of radiotherapy for non-small cell lung carcinoma. *Int J Radiat Oncol Biol Phys* **34**: 315–322

Lindsley KL, Cho P, Stelzer KJ, Koh WJ, Austin Seymour M, Russell KJ, Laramore GE and Griffin TW (1996) Clinical trials of neutron radiotherapy in the United States. *Bull Cancer Radiother* **83**(suppl.): 78–86

Perez CA, Pajak TF, Rubin P, Simpson JR, Mohiuddin M, Brady LW, Perez Tamayo R and Rotman M (1987) Long-term observations of the patterns of failure in patients with unresectable non-oat cell carcinoma of the lung treated with definitive radiotherapy. Report by the Radiation Therapy Oncology Group. *Cancer* **59**: 1874–1881

Saunders MI & Dische S (1990) Continuous, hyperfractionated, accelerated radiotherapy (CHART) in non-small cell carcinoma of the bronchus. *Int J Radiat Oncol Biol Phys* **19**: 1211–1215

Saunders MI, Anderson P, Dische S and Martin WM (1982) A controlled clinical trial of misonidazole in the radiotherapy of patients with carcinoma of the bronchus. *Int J Radiat Oncol Biol Phys* **8**: 347–350

Saunders MI, Bennett MH, Dische S and Anderson, PJ (1984) Primary tumor control after radiotherapy for carcinoma of the bronchus. *Int J Radiat Oncol Biol Phys* **10**: 499–501

Saunders MI, Dische S, Barrett A, Parmar MKB, Harvey A and Gibson D (1997) Continuous hyperfractionated accelerated radiotherapy (CHART) versus conventional radiotherapy in non-small-cell lung cancer: a randomised multicentre trial. *Lancet* **350**: 161–165

Simpson JR, Bauer M, Perez CA, Wasserman TH, Emami B, Doggett RL, Byhardt RW, Phillips TL and Mowry PA (1989) Radiation therapy alone or combined with misonidazole in the treatment of locally advanced non-oat cell lung cancer: report of an RTOG prospective randomized trial. *Int J Radiat Oncol Biol Phys* **16**: 1483–1491

Slevin NJ, Hendry JH, Roberts SA and Agren Cronqvist A (1992) The effect of increasing the treatment time beyond three weeks on the control of T2 and T3 laryngeal cancer using radiotherapy. *Radiother Oncol* **24**: 215–220

Wilson GD (1991) Assessment of human tumour proliferation using bromodeoxyuridine – current status. *Acta Oncol* **30**: 903–910

Withers HR, Taylor JM and Maciejewski B (1988) The hazard of accelerated tumor clonogen repopulation during radiotherapy. *Acta Oncol* **27**: 131–146

© Cancer Research Campaign 1998